# Persisting neuroendocrine abnormalities and their association with physical impairment 5 years after critical illness

**DOI:** 10.1186/s13054-021-03858-1

**Published:** 2021-12-16

**Authors:** Ilse Vanhorebeek, Inge Derese, Jan Gunst, Pieter J. Wouters, Greet Hermans, Greet Van den Berghe

**Affiliations:** 1 Laboratory of Intensive Care Medicine, Department of Cellular and Molecular Medicine, KU Leuven, Herestraat 49, 3000 Leuven, Belgium; 2 Department of Intensive Care Medicine, University Hospitals Leuven, Herestraat 49, 3000 Leuven, Belgium; 3 Clinical Division and Laboratory of Intensive Care Medicine, Department of Cellular and Molecular Medicine, KU Leuven, Herestraat 49, 3000 Leuven, Belgium; 4 Medical Intensive Care Unit, Department of General Internal Medicine, University Hospitals Leuven, Herestraat 49, 3000 Leuven, Belgium

**Keywords:** Critical illness, Long-term, Thyroid axis, Somatotropic axis, Adrenal axis, Physical function

## Abstract

**Background:**

Critical illness is hallmarked by neuroendocrine alterations throughout ICU stay. We investigated whether the neuroendocrine axes recover after ICU discharge and whether any residual abnormalities associate with physical functional impairments assessed 5 years after critical illness.

**Methods:**

In this preplanned secondary analysis of the EPaNIC randomized controlled trial, we compared serum concentrations of hormones and binding proteins of the thyroid axis, the somatotropic axis and the adrenal axis in 436 adult patients who participated in the prospective 5-year clinical follow-up and who provided a blood sample with those in 50 demographically matched controls. We investigated independent associations between any long-term hormonal abnormalities and physical functional impairments (handgrip strength, 6-min walk distance, and physical health-related quality-of-life) with use of multivariable linear regression analyses.

**Results:**

At 5-year follow-up, patients and controls had comparable serum concentrations of thyroid-stimulating hormone, thyroxine (T_4_), triiodothyronine (T_3_) and thyroxine-binding globulin, whereas patients had higher reverse T_3_ (rT_3_, *p* = 0.0002) and lower T_3_/rT_3_ (*p* = 0.0012) than controls. Patients had comparable concentrations of growth hormone, insulin-like growth factor-I (IGF-I) and IGF-binding protein 1 (IGFBP1), but higher IGFBP3 (*p* = 0.030) than controls. Total and free cortisol, cortisol-binding globulin and albumin concentrations were comparable for patients and controls. A lower T_3_/rT_3_ was independently associated with lower handgrip strength and shorter 6-min walk distance (*p* ≤ 0.036), and a higher IGFBP3 was independently associated with higher handgrip strength (*p* = 0.031).

**Conclusions:**

Five years after ICU admission, most hormones and binding proteins of the thyroid, somatotropic and adrenal axes had recovered. The residual long-term abnormality within the thyroid axis was identified as risk factor for long-term physical impairment, whereas that within the somatotropic axis may be a compensatory protective response. Whether targeting of the residual abnormality in the thyroid axis may improve long-term physical outcome of the patients remains to be investigated.

*Trial registration* ClinicalTrials.gov: NCT00512122, registered on July 31, 2007 (https://www.clinicaltrials.gov/ct2/show/NCT00512122).

**Graphical Abstract:**

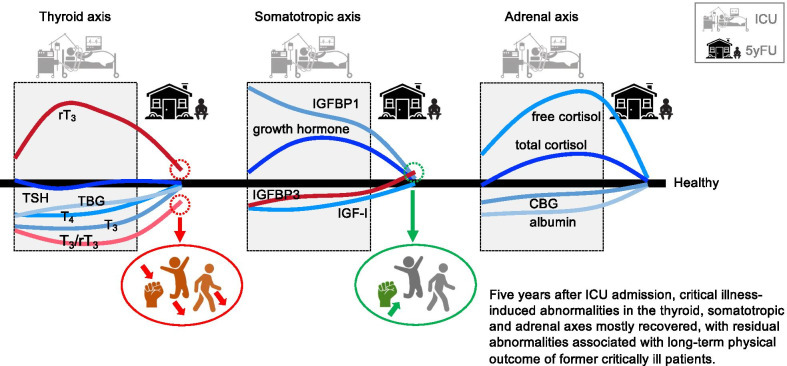

**Supplementary Information:**

The online version contains supplementary material available at 10.1186/s13054-021-03858-1.

## Background

Critical illness is hallmarked by pronounced neuroendocrine alterations, with those within the thyroid axis, the somatotropic axis and the adrenal axis most extensively studied [1]. The typical responses follow a biphasic pattern, distinguishing between the acute and prolonged phase of critical illness [1, 2]. In the acute phase (first hours to days), the anterior pituitary is actively secreting hormones, with a transient rise in thyroid-stimulating hormone (TSH) and an increase in growth hormone [1–5]. An acute rise in adrenocorticotropic hormone (ACTH) has been described in patients with sepsis or multiple-trauma [6], but such rise was not observed in heterogeneous general ICU patients [7, 8]. The active pituitary hormone secretion occurs in the face of altered peripheral hormone metabolism, altered target organ sensitivity, and altered hormone binding proteins [4–13]. These alterations reduce the availability of most anabolic effector hormones, including triiodothyronine (T_3_) and insulin-like growth factor-I (IGF-I), while the availability of the catabolic stress hormone cortisol increases. When illness is prolonged beyond the first few days, the neuroendocrine axes are uniformly suppressed, with low target organ hormone levels or, in case of cortisol, insufficiently elevated or normal levels [1, 2, 5, 7, 10, 14]. This suppression during the prolonged phase of illness is of central/hypothalamic origin [1, 2, 15].

Whereas the neuroendocrine responses in ICU have been well documented, data after ICU discharge are scarce and mostly limited to patients who suffered from brain damage either due to traumatic brain injury (TBI) or brain surgery. Although TBI-associated neuroendocrine disturbances often resolve, persistent hypopituitarism remains present in many patients up to years after the insult, associated with poor recovery and worse long-term outcome (e.g. cognitive impairment, decreased exercise capacity, poor quality-of-life) [16–18]. Likewise, survivors of brain tumors show a high risk of hypopituitarism and need for hormone replacement therapy many years later [19, 20]. Heterogeneous prolonged critically ill adult patients showed a uniform rise in ACTH and cortisol to supra-normal levels from ICU discharge to one week later [14]. In children, salivary cortisol levels were normal months to years after ICU admission [21, 22]. In the absence of other data, it remained unclear whether the neuroendocrine abnormalities that are present in general adult ICU patients recover in the long-term.

In this study of patients who were followed-up 5 years after ICU admission for heterogeneous diagnoses, we compared hormonal parameters within the thyroid axis, the somatotropic axis and the adrenal axis with those of demographically matched controls, and investigated whether any long-term neuroendocrine abnormalities in former ICU patients associate with long-term physical functional impairments.

## Methods

### Study design and participants

This is a preplanned secondary analysis of patients included in the EPaNIC study and its long-term follow-up. The EPaNIC study randomly allocated 4640 adult critically ill patients who were nutritionally at risk (score of 3 or more on the Nutritional Risk Screening scale) to initiation of supplemental parenteral nutrition (PN) completing insufficient enteral nutrition (EN) within 48 h (early-PN), or to withholding of supplemental PN in the first week of intensive care (late-PN) [23]. All patients received EN as soon as possible, insulin infusions to maintain normoglycemia, and parenteral trace elements, minerals and vitamins. Patients were not eligible for participation in the EPaNIC study if younger than 18 years, if moribund or coded “do not resuscitate”, if enrolled in another trial, if suffering from short-bowel syndrome, if on home ventilation, if in diabetic coma, if referred with nutritional regimen, if pregnant or lactating, if not having a central catheter, if taking oral nutrition, if readmitted to ICU, or if BMI was lower than 17 kg/m^2^.

**Fig. 1:**
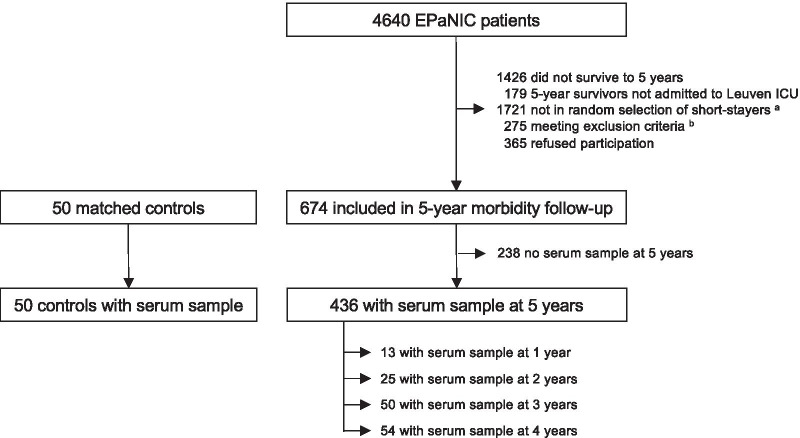
Flowchart of study participants and study design. The major focus of this study is on the patients who participated in the 5-year morbidity follow-up of the EPaNIC study and for whom a serum sample had been collected at this time point. A small subgroup of these patients had also participated at one or more earlier time points, 1-, 2-, 3- or 4-years post-ICU. ^a^For feasibility reasons only a random subset of short-stay patients in ICU for fewer than 8 days were eligible for the 5-year EPaNIC follow-up study [24]. The subgroup of short-stay patients was a random, computer-generated “3 out of 10” sample, weighed within admission diagnostic categories to a distribution similar to that among long-stay patients in ICU for at least 8 days (who all were eligible). Of the short-stay patients, 1721 were not in that random selection. The total 5-year follow-up cohort consisted of 398 short-stay and 276 long-stay patients. ^b^Of the eligible patients, 275 were subsequently excluded for meeting one or more exclusion criteria. These were patients with pre-ICU neuromuscular disorders, unable to walk without assistance prior to ICU or other disabilities present before follow-up potentially confounding morbidity endpoints (i.e. cardiac assist device, pulmonary resection, psychiatric disease, dementia, vegetative state, in hospital/rehabilitation center/nursing home); patients who could not be not contacted; patients who died after five years post-ICU before the planned testing; patients for whom the time window had passed (predefined time window for the 5-year follow-up had been set at 5 ± 0.5 years after ICU admission); or patients for whom there was a language barrier [24]. ICU: intensive care unit

A subgroup of the patients had been assessed for long-term morbidity, 5 years after ICU admission, during hospital or home visits [24]. For that follow-up study, all long-stay patients in ICU for at least 8 days were eligible, whereas for feasibility reasons only a random subset of short-stay patients in ICU for fewer than 8 days were eligible [24]. The subgroup of short-stay patients was a random, computer-generated “3 out of 10” sample, weighed within admission diagnostic categories to a distribution similar to that among long-stay patients. Patients suffering from conditions that could confound the morbidity endpoints had been excluded (*n* = 128). Such conditions comprised pre-existing neuromuscular disorders or inability to walk without assistance before ICU admission, or other physical disabilities present before follow-up potentially confounding morbidity endpoints (cardiac assist device, pulmonary resection, psychiatric disease, dementia, vegetative state, in hospital/rehabilitation center/nursing home). The total 5-year follow-up cohort consisted of 674 patients, among which 398 short-stay and 276 long-stay patients (Fig. 1). As controls, 50 individuals had been recruited via primary care givers and outpatient clinics, with the only exclusion criteria being having required an ICU admission or suffering from conditions that could confound the morbidity endpoints (i.e. neuromuscular disorders, inability to walk or other physical disabilities).

Of the patients who participated in the 5-year follow-up study, 436 provided a blood sample during the follow-up visit, among which 265 with a short-stay and 171 with a long-stay in ICU (Fig. 1). All 50 controls had provided a blood sample. Some patients had also participated and donated a blood sample in earlier follow-up studies (1-year follow-up: *n* = 13, 2-year follow-up: *n* = 25, 3-year follow-up: *n* = 50, 4-year follow-up: *n* = 54). Serum was extracted from the blood samples and stored at − 80 °C.

Institutional Review Board approval of the study and of the consent forms was obtained (ML4190). All patients or their next-of-kin provided written informed consent for participation in the EPaNIC study and all former ICU patients and all controls provided written informed consent for participation in the follow-up study. The EPaNIC study protocol and primary results on short- and long-term outcomes of the participating patients have been published [23–27].

### Serum analyses

Serum concentrations of hormones and binding proteins were measured with commercially available immunoradiometric assay (IRMA), radioimmunoassay (RIA), enzyme-linked immunosorbent assay (ELISA) or colorimetric quantification. The thyroid axis was evaluated via quantification of TSH (IRMA IM3712, Beckman Coulter, Prague, Czech Republic), total tetraiodothyronine (T_4_, RIA IM1447, Beckman Coulter), total T_3_ (RIA IM1699, Beckman Coulter), reverse T_3_ (rT_3_, RIA R-EW-125, RIAZEN, ZenTech s.a., Liège, Belgium), T_3_/rT_3_ and thyroxine-binding globulin (TBG, ELISA LS-F4616, LifeSpan Biosciences, Seattle, WA), the somatotropic axis via growth hormone (RIA KIP1081, DIAsource ImmunoAssays S.A., Louvain-la-Neuve, Belgium), IGF-I (ELISA DG100, R&D Systems, Abingdon, UK, assay previously validated against acid gel filtration for extraction of potentially interfering binding proteins [5]), IGF-binding protein 3 (IGFBP3, ELISA DGB300, R&D Systems) and IGFBP1 (ELISA KAPME01, DIAsource ImmunoAssays S.A), and the adrenal axis via total cortisol (RIA IM1841, Beckman Coulter), cortisol-binding globulin (CBG, RIA R-AJ-100, RIAZEN, ZenTech s.a.), albumin (colorimetric assay bromocresol-green method MAK-124, Sigma-Aldrich, Bornem, Belgium) and calculated free cortisol. Free cortisol was calculated with the Coolens formula, adapted for individual CBG and albumin, validated in the ICU context [13, 28]), with free cortisol (in µmol/l) = square root (((0.0167 + (*G* − *T*)/(2 * (1 + *N*″)))^2^ + *T*/((1 + *N*″) * *K*)))  − 0.0167 + (*G* − *T*)/(2 * (1 + *N*″)), where *G* = plasma CBG concentration (in µmol/l), *T* = plasma total cortisol concentration (in µmol/l), *K* = affinity of CBG for cortisol = 3.10^7^ M^−1^, and *N*″ = 1.74/43 × individual albumin concentration (in g/l). Free cortisol was converted from µmol/l to µg/dl by multiplying by 1000 and dividing by 27.59.

Apart from the serum samples available for the patients at 5-year follow-up and at the intermediary follow-up moments, we also investigated all patient serum samples available upon ICU admission, at day 4 and 7 if still in ICU, and at the last ICU day to document time series up until the 5-year follow-up.

### Measures of physical function

At 5-year follow-up, several measures of physical functional capacity had been evaluated [24, 25]. Handgrip strength was measured with a hydraulic handgrip dynamometer (Jamar Preston, Jackson, MI), with values expressed as percent of predicted values for sex and age. The 6-min walk distance was used as a measure of exercise capacity, expressed as percent of predicted values taking into account sex, age, height and weight, and with imputation of a zero-value for patients unable to perform the test due to physical limitations. Physical functioning was assessed with the physical component score (PCS) of the Medical Outcomes Report–Short Form 36 (SF36) health-related quality-of-life questionnaire (score range 0–100, higher values indicating better performance).

### Statistical analyses

Characteristics and physical outcomes of patients and controls are reported as numbers (frequencies) or medians (interquartile ranges), and were compared with Chi-square/Fisher-exact or Mann–Whitney U tests. Outcomes were also studied with multivariable linear regression analysis, adjusted for age, sex, and BMI, reported as *β*-estimates (95% CIs).

The evolution within former ICU patients of the serum concentrations of hormones and binding proteins from the last ICU day to the 5-year follow-up moment was assessed with repeated-measures analysis of variance (ANOVA). The comparison of these hormonal parameters between former ICU patients at 5-year follow-up and matched controls was performed with Student *t* test. For these analyses, not-normally distributed data were transformed to a near-normal distribution (square root or square root-square root transformation as specified in the figure legends). Control subjects or patients who during ICU stay or at follow-up received hormones interfering with the measurements of the hormonal parameters of the respective axes were excluded from these analyses. Values at 1-, 2-, 3- and 4-years post-ICU, for those patients who had donated a sample at the respective time points, were also visualized for illustrative purposes only in view of the small sample sizes. Likewise, values upon ICU admission and on ICU day 4 and 7 were plotted to verify whether the patients showed the typical critical illness-induced neuroendocrine changes during ICU stay, thus assessing representativeness of the cohort.

In exploratory analyses, we investigated whether we could identify illness-associated or post-ICU factors that may independently associate with the concentrations of the hormonal parameters at 5-year follow-up. Therefore, we performed multivariable linear regression analyses adjusting for demographics (sex, age and gender at 5-year follow-up), randomization to late-PN or early-PN, risk of malnutrition, type and severity of illness, a sepsis diagnosis upon ICU admission, duration of critical illness (dichotomized as an ICU stay shorter than 8 days or at least 8 days [24]), history of diabetes or malignancy, medications taken chronically at follow-up, and need for hospital readmission between ICU discharge and follow-up. We also performed a stratified analysis comparing patients with a duration of critical illness shorter than 8 days or at least 8 days in univariable analyses and in multivariable analyses adjusted for demographics.

To assess whether any long-term neuroendocrine abnormalities are independently associated with physical impairments in ICU survivors at 5-year follow-up, we performed multivariable linear regression analyses among the former ICU patients, entering the hormones or binding proteins that were different for patients and controls at 5-year follow-up as variables in the models, adjusting for age, sex, and BMI. Patients who at the time of follow-up received hormone treatments interfering with the measurements for the respective axes were excluded from these analyses.

Statistical analyses were performed with JMP®Pro15.1.0 (SAS-Institute, Cary, NC). Two-sided *p* values < 0.05 were considered to indicate statistical significance. No corrections for multiple comparisons were done.

## Results

Age, sex and BMI distributions of the 436 patients who participated in the 5-year follow-up study were similar to those of the 50 controls (Table 1). Patient characteristics upon ICU admission and ICU outcomes are shown in Table 2. This study obviously focused on a subgroup of survivors, who were overall younger, had fewer comorbidities, were less severely ill upon ICU admission, and showed fewer complications during ICU stay as compared with non-surviving patients (Additional file 1: Table S1). Inherent to the study design, the studied patient cohort was relatively enriched in long-stay patients when compared with the original EPaNIC cohort [23], thus presenting with more severe illness upon ICU admission and suffering from more complications during ICU stay as compared with the cohort of other surviving patients (Additional file 2: Table S2). Among the participants in the 5-year physical outcome study [24], blood samples were only drawn from patients who were able to come to the hospital, who were younger and had fewer comorbidities than those who were examined at home (Additional file 3: Table S3).

**Table 1:** Participants’ demographics and physical function at 5-year follow-up

Characteristic	Patients (*n* = 436)	Controls (*n* = 50)	*p*
Demographics at 5-year follow-up			
Male sex, no (%)	302 (69.3)	35 (70.0)	0.91
Age (years), median (IQR)	61 (51–69)	61 (57–66)	0.51
BMI (kg/m^2^), median (IQR)	27.3 (24.2–30.5)	26.4 (23.7–29.3)	0.30
Physical function at 5-year follow-up			
Univariable analysis			
Handgrip strength dominant hand (%pred), median (IQR)	93 (78–107)	104 (91–119)	< 0.0001
Handgrip strength non-dominant hand (%pred), median (IQR)	100 (83–117)	114 (102–125)	0.0002
6-min walk distance (%pred), median (IQR)	95 (80–106)	117 (107–125)	< 0.0001
Physical component score SF36, median (IQR)	48 (38–55)	55 (51–58)	< 0.0001
Multivariable analysis^a^			
Handgrip strength dominant hand (%pred), *β*-estimate (95% CI)	− 6.194 (− 9.369; − 3.019)	0.0001	
Handgrip strength non-dominant hand (%pred), *β*-estimate (95% CI)	− 5.401 (− 8.962; − 1.840)	0.0030	
6-min walk distance (%pred), *β*-estimate (95% CI)	− 11.151 (− 14.212; − 8.091)	< 0.0001	
Physical component score SF36, *β*-estimate (95% CI)	− 3.781 (− 5.326; − 2.235)	< 0.0001	

^a^*β*-estimates are indicated for patients versus controls

*%pred* percent of predicted, *CI* confidence interval, *IQR* interquartile range, *SF36* Medical Outcomes Report–Short Form 36

**Table 2:** Patient characteristics at the time of critical illness

Characteristic	Patients (*n* = 436)
Characteristics upon ICU admission	
Age (years), median (IQR)	56 (46–64)
BMI (kg/m^2^), median (IQR)	25.7 (23.1–28.4)
Randomized to early PN, no (%)	222 (50.9)
Nutritional risk score ≥ 5, no (%)	70 (16.1)
APACHE-II score first 24 h, median (IQR)	26 (16–33)
Emergency admission, no (%)	271 (62.2)
Admission diagnosis, no (%)	
Cardiac surgery	168 (38.5)
Complicated abdominal or pelvic surgery	44 (10.1)
Transplantation	76 (17.4)
Trauma, burns or reconstructive surgery	57 (13.1)
Complicated pulmonary or esophageal surgery	12 (2.8)
Respiratory disease	9 (2.1)
Complicated vascular surgery	19 (4.4)
Gastroenterologic or hepatic disease	9 (2.1)
Complicated neurosurgery	16 (3.7)
Hematological or oncological disease	2 (0.5)
Neurological disease	2 (0.5)
Cardiovascular disease	3 (0.7)
Renal disease	1 (0.2)
Neurological presentation of medical disease	2 (0.5)
Metabolic disorder	1 (0.2)
Other	15 (3.4)
History of diabetes, no (%)	47 (10.8)
History of malignancy, no (%)	55 (12.6)
Pre-admission dialysis, no (%)	3 (0.7)
Sepsis upon admission, no (%)	118 (27.1)
ICU outcomes	
New infection in ICU, no (%)	145 (33.3)
New need of dialysis, no (%)	38 (8.7)
Duration of mechanical ventilation (days), median (IQR)	3 (1–8)
Corticosteroid treatment, no (%)	148 (33.9)
Duration of corticosteroid treatment (days), median (IQR)	0 (0–2)
ICU length of stay (days), median (IQR)	5 (2–13)
Hospital length of stay (days), median (IQR)	21 (11–35)

*APACHE-II score* acute physiology and chronic health evaluation-II score, *ICU* intensive care unit, *IQR* interquartile range, *PN* parenteral nutrition

### Five-year impact of critical illness requiring ICU admission on the neuroendocrine axes

#### Thyroid axis

**Fig. 2:**
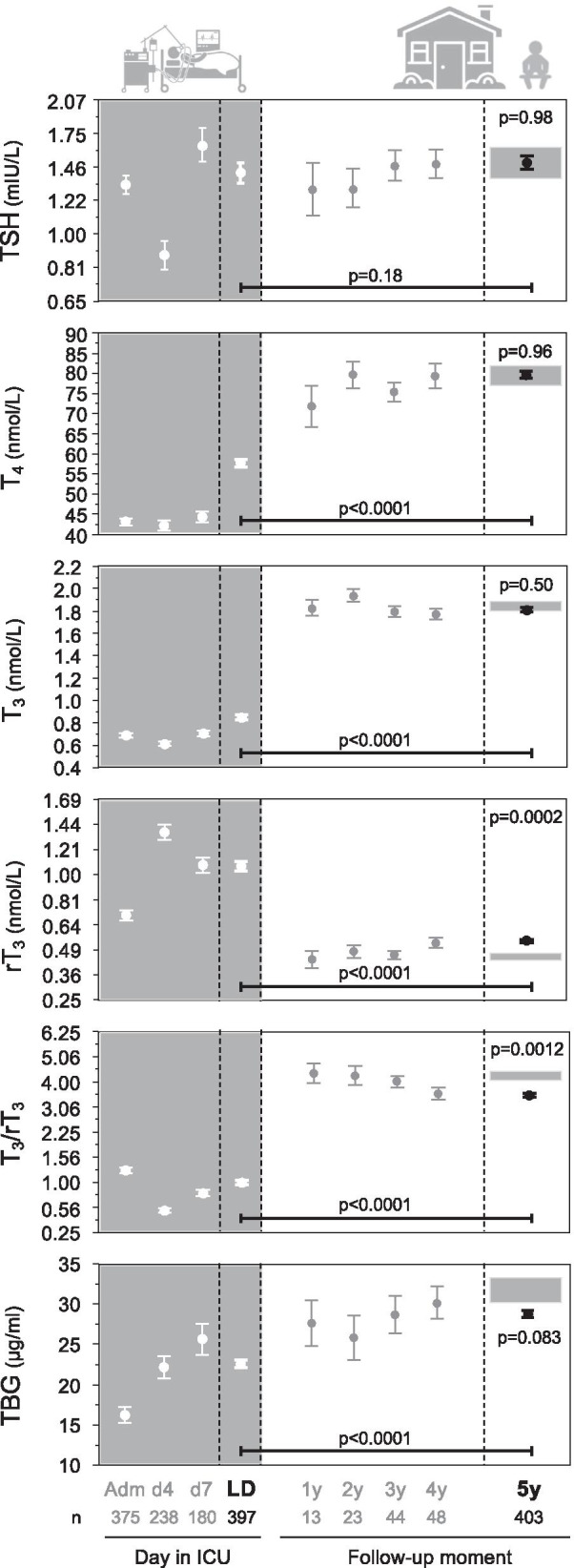
Thyroid axis 5 years after ICU admission: comparison with controls and within-patient evolution from ICU discharge. Data are shown as mean and standard error of the mean. The gray rectangles at the right side of the panels reflect mean plus or minus the standard error of the mean of the controls matched to the patients at 5-year follow-up. TSH concentrations were square root-square root transformed and rT_3_ and T_3_/rT_3_ were square root transformed to obtain a near normal distribution, allowing repeated-measures ANOVA and *t* test. *Y*-axes were transformed back to original values. Patients who received thyroid hormone treatment in ICU or were on chronic thyroid hormone treatment at follow-up were excluded. TBG concentrations were measured in all available LD and 5y samples, but only in a subset of the samples available for the other time points (Adm: *n* = 34, d4: *n* = 44, d7: *n* = 33, 1y: *n* = 12, 2y: *n* = 11, 3y: *n* = 18, 4y: *n* = 18). Adm: ICU admission, d4: day 4 in ICU, d7: day 7 in ICU, LD: last day in ICU, 1y: one year after ICU admission, 2y: two years after ICU admission, 3y: three years after ICU admission, 4y: four years after ICU admission, 5y: five years after ICU admission, ICU: intensive care unit

Thirty-three patients and 2 controls treated with thyroid hormone in ICU or at follow-up were excluded for this analysis. Of the excluded patients, 3 received thyroid hormone treatment only in ICU, 15 were on thyroid treatment in ICU and at follow-up and the other 15 only at follow-up. During ICU stay, the studied patients revealed the typical low-normal serum TSH and low T_4_, T_3_ and TBG concentrations, whereas rT_3_ was high, resulting in a low T_3_/rT_3_ ratio (Fig. 2). When compared with the last ICU day, TSH concentrations of former ICU patients assessed at 5-year follow-up had remained stable, whereas T_4_ and T_3_ had increased, rT_3_ had decreased, and T_3_/rT_3_ and TBG had increased (*p* < 0.0001). The hormonal concentrations documented at 5-year follow-up were roughly already reached at earlier follow-up time points 1 to 4 year after ICU admission. At 5-year follow-up, serum concentrations of TSH, T_4_, T_3_ and TBG in former ICU patients were comparable to those in controls. In contrast, rT_3_ was higher (*p* = 0.0002) in former ICU patients than in controls, with rT3 concentrations even being higher than the upper normal reference range for 46.3% of the patients. Consequently, T_3_/rT_3_ (*p* = 0.0012) was lower in former ICU patients than in controls.

#### Somatotropic axis

**Fig. 3:**
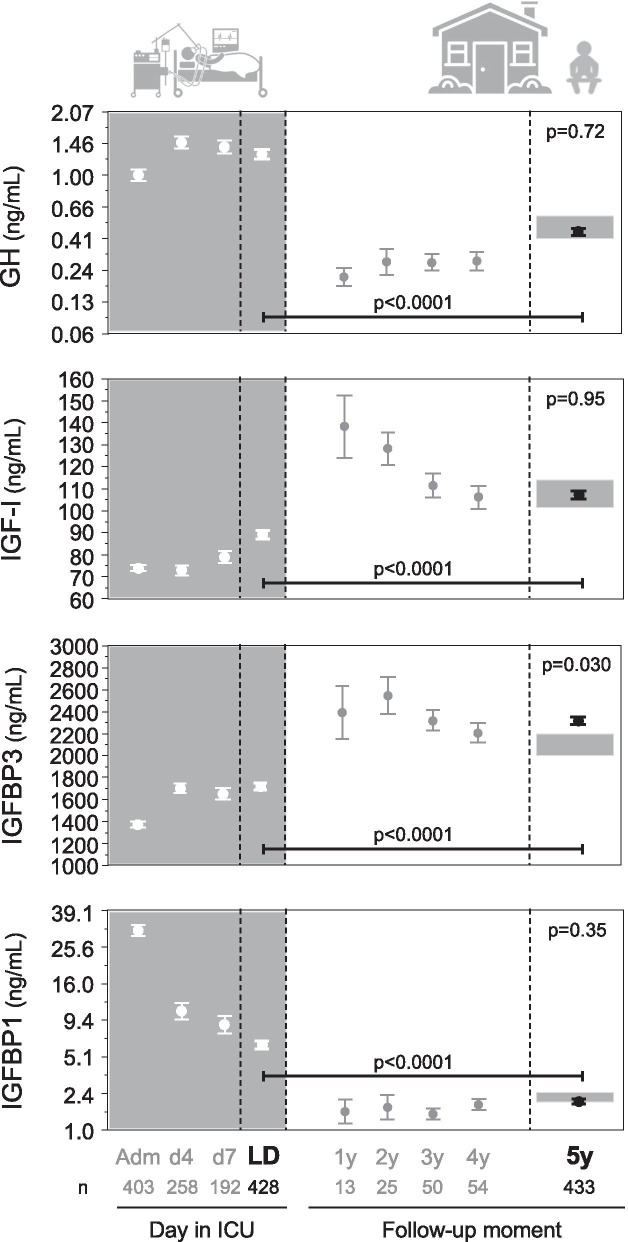
Somatotropic axis 5 years after ICU admission: comparison with controls and within-patient evolution from ICU discharge. Data are shown as mean and standard error of the mean. The gray rectangles at the right side of the panels reflect mean plus or minus the standard error of the mean of the controls matched to the patients at 5-year follow-up. Growth hormone and IGFBP1 concentrations were square root-square root transformed to obtain a near normal distribution, allowing repeated-measures ANOVA and *t* test. *Y*-axes were transformed back to original values. Patients on chronic GHRH or somatostatin analogue treatment at follow-up were excluded. Adm: ICU admission, d4: day 4 in ICU, d7: day 7 in ICU, LD: last day in ICU, 1y: one year after ICU admission, 2y: two years after ICU admission, 3y: three years after ICU admission, 4y: four years after ICU admission, 5y: five years after ICU admission, ICU: intensive care unit

Three patients on chronic growth hormone-releasing hormone (GHRH) or somatostatin analogue treatment at follow-up were excluded for this analysis. During ICU stay, the studied patients revealed the typical high concentrations of growth hormone and IGFBP1 and low IGF-I and IGFBP3 concentrations (Fig. 3). From the last ICU day toward the 5-year follow-up, growth hormone and IGFBP1 had decreased, and IGF-I and IGFBP3 had increased (*p* < 0.0001). Mostly, concentrations observed at 5-year follow-up were already reached at earlier follow-up time points 1 to 4 year after ICU admission. At 5-year follow-up, former ICU patients had similar serum concentrations of growth hormone, IGF-I and IGFBP1, but higher IGFBP3 concentrations (*p* = 0.030) than the control group, with 3.2% of patients even exceeding the upper normal reference range of IGFBP3.

#### Adrenal axis

**Fig. 4:**
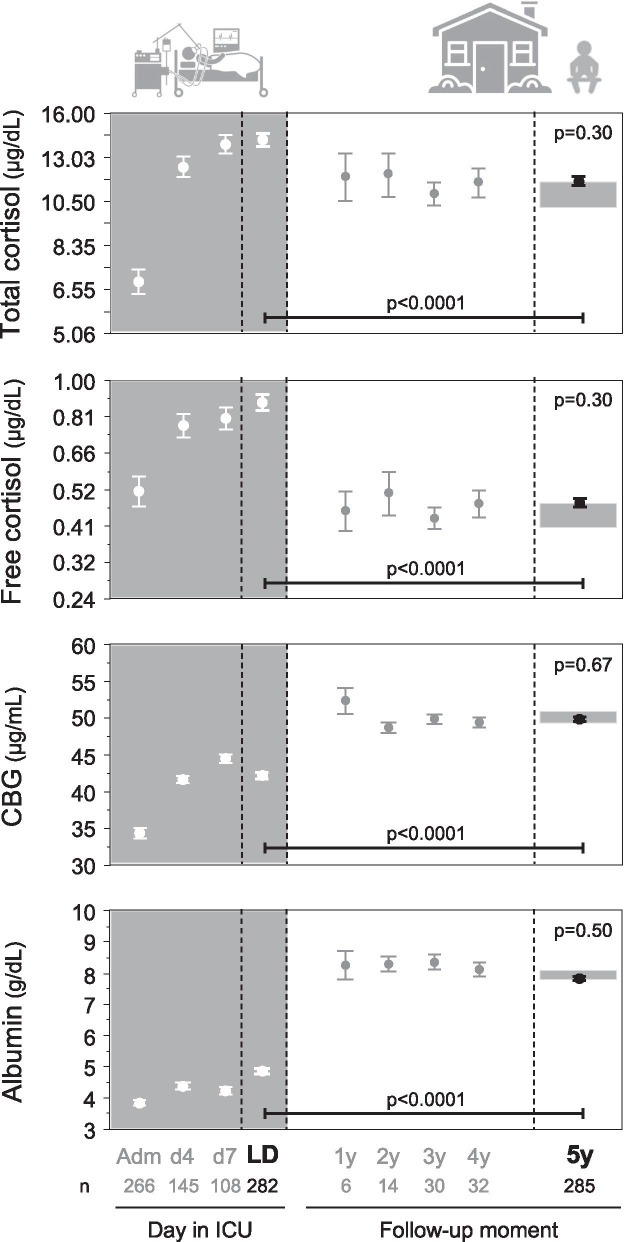
Adrenal axis 5 years after ICU admission: comparison with controls and within-patient evolution from ICU discharge. Data are shown as mean and standard error of the mean. The gray rectangles at the right side of the panels reflect mean plus or minus the standard error of the mean of the controls matched to the patients at 5-year follow-up. Total and free cortisol concentrations were square root-square root transformed to obtain a near normal distribution, allowing repeated-measures ANOVA and *t* test. *Y*-axes were transformed back to original values. Patients on corticosteroid treatment in ICU or on chronic corticosteroid treatment at follow-up were excluded. Adm: ICU admission, d4: day 4 in ICU, d7: day 7 in ICU, LD: last day in ICU, 1y: one year after ICU admission, 2y: two years after ICU admission, 3y: three years after ICU admission, 4y: four years after ICU admission, 5y: five years after ICU admission, ICU: intensive care unit

Patients on corticosteroid treatment in ICU or at follow-up (*n* = 151) were excluded for this analysis. Of the excluded patients, 104 received corticosteroid treatment only in ICU, 44 were on corticosteroid treatment in ICU and at follow-up and the other 3 only at follow-up. The studied patients revealed the typical high total and free cortisol and low CBG and albumin concentrations beyond the ICU admission day until ICU discharge (Fig. 4). From the last ICU day toward 5-year follow-up of former ICU patients, total and free cortisol concentrations had decreased, whereas CBG and albumin concentrations had increased (*p* < 0.0001), with similar changes already present at earlier follow-up time points 1 to 4 year after ICU admission. At 5-year follow-up, serum total and free cortisol, CBG and albumin concentrations of former ICU patients were comparable to those of controls.

### Factors associated with hormonal parameters at 5-year follow-up

Some occasional associations were found with type of critical illness requiring ICU admission or medications taken at follow-up (Additional file 4: Table S4). Severity of illness was only associated with growth hormone and IGFBP1 concentrations at follow-up. Sepsis upon admission, randomization to timing of initiating supplemental parenteral nutrition in ICU, or prolonged need of intensive care were not independently associated with any of the hormonal parameters. Also a stratified analysis for duration of critical illness did not reveal any significant difference in the hormonal parameters of patients who depended on intensive care for less than 8 days or at least 8 days (Additional file 5: Fig. S1).

### Association of residual neuroendocrine abnormalities at 5-year follow-up with physical function

As documented for the original patient cohort [24], also the presently studied subgroup of former ICU patients with a serum sample available at 5-year follow-up showed long-term impairment of physical function as compared with matched controls, as evidenced by less handgrip strength, shorter 6-min walk distance, and worse self-reported physical quality-of-life as revealed by the SF36 PCS, both in univariable and multivariable analyses adjusted for age, sex and BMI (Table 1).

Since we showed that T_3_/rT_3_ and IGFBP3 in patients at 5-year follow-up were different from controls, we next investigated whether T_3_/rT_3_ and IGFBP3 associated with the 5-year physical impairments of the former ICU patients, after exclusion of patients on chronic treatment with thyroid hormone, GHRH or somatostatin analogues (Table 3). Among former ICU patients, a lower T_3_/rT_3_ ratio at 5-year follow-up was independently associated with less handgrip strength for dominant (*p* = 0.036) and non-dominant hand (p = 0.030) and with a shorter 6-min walk distance (*p* = 0.014). More specifically, for every standard deviation decrease in T_3_/rT_3_, grip strength decreased with 2.4 kg for the dominant hand and with 2.8 kg for the non-dominant hand, and the 6-min walk distance decreased with 3 m. A lower T_3_/rT_3_ ratio was not independently associated with worse physical quality-of-life (*p* = 0.13). A higher IGFBP3 concentration at 5-year follow-up was independently associated with more handgrip strength for the dominant hand (*p* = 0.031), with an increase in grip strength with 2.2 kg for every standard deviation increase in IGFBP3. The IGFBP3 concentration was not independently associated with the other functional outcomes.

**Table 3:** Association of long-term neuroendocrine abnormalities with long-term physical function 5-years after critical illness

Outcome	*β*-estimate (95% CI)	*p*
Handgrip strength dominant hand		
T3/rT3 *Z*-score per unit added	2.372 (0.153; 4.592)	0.036
IGFBP3 *Z*-score per unit added	2.158 (0.191; 4.126)	0.031
Handgrip strength non-dominant hand		
T3/rT3 *Z*-score per unit added	2.758 (0.257; 5.259)	0.030
IGFBP3 *Z*-score per unit added	1.093 (− 1.138; 3.324)	0.33
6-min walk distance		
T3/rT3 *Z*-score per unit added	2.746 (0.551; 4.941)	0.014
IGFBP3 *Z*-score per unit added	− 1.675 (− 3.625; 0.275)	0.092
Physical component score SF36		
T3/rT3 *Z*-score per unit added	0.905 (− 0.289; 2.098)	0.13
IGFBP3 *Z*-score per unit added	− 0.277 (− 1.311; 0.758)	0.59

Patients on chronic thyroid hormone, GHRH or somatostatin analogue treatment were excluded for these analyses. Models were adjusted for sex, and age and BMI at 5-year follow-up

*CI* confidence interval, *SF36* Medical Outcomes Report–Short Form 36

## Discussion

Patients admitted to the ICU develop typical neuroendocrine changes within the thyroid axis, the growth hormone axis and the adrenal axis in response to critical illness, with pronounced disturbances remaining present until the day of ICU discharge. In this study, we demonstrated that 5 years after critical illness, most of these neuroendocrine abnormalities had normalized, with the exception of rT_3_ concentrations that remained supranormal, resulting in persistently low T_3_/rT_3_ ratios, and of IGFBP3 concentrations that rose from subnormal levels in the ICU to supranormal levels at 5-year follow-up. The lower T_3_/rT_3_ ratios observed in former ICU patients were independently associated with several measures of worse long-term physical capacity, whereas the higher IGFBP3 concentrations were independently associated with better physical capacity (handgrip strength).

Studies investigating neuroendocrine function after recovery from critical illness outside the setting of TBI or brain surgery are scarce if not absent. It has been shown that former ICU patients who were transferred to long-term care facilities still reveal the typical non-thyroidal illness syndrome, which is not unexpected given that such patients have not fully recovered [29]. However, information on the thyroid axis in fully recovered former ICU patients was hitherto lacking. We here observed normal serum TSH, T_4_, T_3_, and TBG concentrations 5 years after ICU admission, whereas rT_3_ remained elevated, resulting in an abnormally low T_3_/rT_3_ ratio. The underlying mechanism of this long-term abnormality within the thyroid axis remains unclear, but could involve persisting changes in the expression or activity of the deiodinases that control the conversion of T_4_ to T_3_ and rT_3_. We also demonstrated that in the former ICU patients assessed 5 years later, serum growth hormone, IGF-I and IGFBP1 had normalized, whereas IGFBP3 was increased reaching supranormal levels. Increases in IGF-I and IGFBP3 (and T_4_) have also been documented over a 2-year time-period in children after severe burn injury, after they had acutely dropped in the critical phase of the injury, but comparison with healthy children was not performed [30]. IGFBP3 is a binding protein that positively regulates tissue availability of IGF-I [31]. Its expression is regulated by growth hormone, insulin, androgens, and vitamin D, among others, with involvement of DNA methylation and transcriptional, posttranscriptional and translational control [32]. Whether alterations in these regulators play a role in the supranormal IGFBP3 levels of former ICU patients remains unknown. Although we have previously shown that 1 week after ICU discharge of prolonged critically ill adult patients ACTH and cortisol levels had risen to supranormal levels [14], the current data suggest that the adrenal axis recovers thereafter. This finding is consistent with normal salivary cortisol levels observed in former critically ill children, assessed months to years after pediatric-ICU admission [21, 22]. Interestingly, however, in acute-respiratory-distress-syndrome survivors, an inverse correlation has been reported between long-term basal serum cortisol level and increasing traumatic ICU memories [33]. Unlike in human patients, long-term perturbations of the adrenal axis have been observed in rodent models of sepsis. In mice, increased stress-induced corticosterone and increased adrenal weights have been observed 2 to 7 weeks after induction of sepsis via cecal ligation and puncture [34]. In rats injected with lipopolysaccharide, desensitization of the adrenal axis has been described weeks later [35].

Observing the residual long-term neuroendocrine changes evidently raises questions about their physiological relevance. Reverse T_3_ has long been considered an inactive metabolite of thyroid hormone, considering its weak affinity for nuclear thyroid hormone receptors [36]. Recently, however*, *in vitro studies have suggested an active role for rT_3_ through interaction with extranuclear receptors, though physiological relevance hereof remains to be established [36]. In ICU, higher rT_3_ and lower T_3_/rT_3_ ratios have been associated with worse outcome of critically ill patients [37, 38]. In a rat stroke model, however, a protective effect of rT_3_ has been suggested [39]. Altered IGFBP3 levels may affect IGF-I transport, bioavailability and activity [31]. However, also pleiotropic IGF-independent actions of IGFBP3 have been described, regulating gene transcription with effects on cell growth, survival and apoptosis [31]. To explore any potential physiological relevance of the documented long-term neuroendocrine abnormalities in former ICU patients, we studied associations with long-term physical function. Interestingly, we observed an independent association of a persisting low T_3_/rT_3_ ratio with decreased physical performance, including lower handgrip strength and shorter 6-min walk distance. For handgrip strength, the effect size appeared clinically relevant, considering 5 kg as minimal clinically important difference [40, 41] was reached with a two-standard-deviations change in the T_3_/rT_3_ ratio. The effect size for the 6-min walk distance was far below the minimal clinically important difference of 14–30 m [42]. Nevertheless, the combination of all data suggests that the residual abnormality within the thyroid axis may confer an increased risk of long-term physical impairment, and would thus be a harmful long-term consequence of critical illness, at least for a subgroup of patients. Such an interpretation is plausible as thyroid hormone affects diverse aspects of skeletal muscle physiology, being key in the regulation of the muscle’s contractile function, energy metabolism, myogenesis and muscle regeneration [43]. Thus, thyroid hormone action plays an important role in the maintenance of muscle strength and physical functioning. Outside the context of critical illness, patients with newly diagnosed thyroid disease complain about weakness, fatiguability, muscle pain, stiffness and cramps, which usually resolve after treating the thyroid disease [44]. In middle-aged and older euthyroid subjects, a higher free T_3_ level has been independently associated with a higher handgrip strength and physical function, and with an attenuated decline in handgrip strength over time, whereas no association was found for free T_4_ or TSH [45, 46]. Another study evaluating only TSH and free T_4_ found an independent association of low-normal TSH with lower handgrip strength in elderly euthyroid men, but not in post-menopausal women [47]. In young, euthyroid men, rT_3_ has been inversely associated with lean body mass, as have thyroid hormones [48]. In independently living elderly men, high supranormal rT_3_ levels, but also higher free T_4_ levels within the normal range, were independently associated with worse physical performance and lower muscle strength (handgrip, leg extensor), whereas an isolated low T_3_ level remarkably was associated with better physical performance [49]. For IGFBP3, association with long-term physical outcome of former ICU patients was less clear than for T_3_/rT_3_, as such association was only found for handgrip strength. Considering a higher IGFBP3 was associated with better handgrip strength, the supranormal levels of IGFBP3 years after critical illness could be interpreted as a beneficial, compensatory response and thus would not explain long-term physical impairment after critical illness. Positive associations of IGFBP3 with physical outcome in aging have previously been documented for activities of daily living (ADL) in women but not men, for handgrip strength in a cohort of 89-year-old women, and for get-up-and-go times in a mixed gender historical cohort [50, 51]. Most studies in middle-aged to elderly people, however, failed to independently associate IGFBP3 with functional performance measures such as walking speed, grip-strength, or ADL [50, 52–55].

The identification of long-term abnormalities in the thyroid axis as a potential contributor to the long-term physical legacy after critical illness is important, as pathophysiological insight in the long-term physical impairments in ICU survivors is scarce [56–58]. Many prolonged critically ill patients who developed critical illness polyneuropathy showed signs of chronic partial denervation up to 5 years after ICU discharge, whereas persisting evidence of myopathy in patients who developed critical illness myopathy appeared unusual [59, 60]. One small study in prolonged critically ill patients with persistent weakness as assessed 6 months after ICU discharge suggested normalization of proteolysis, autophagy, inflammation and mitochondrial content in muscle, but persistence of impaired regenerative capacity [61, 62]. Studies in mice suggested involvement of sustained mitochondrial dysfunction in chronic sepsis-induced muscle weakness [63], and also showed that engraftment of mesenchymal stem cells improved muscle regeneration and strength after sepsis [64].

This study has limitations to consider. First, we analyzed single samples, whereas several hormones show pulsatile patterns [1, 2]. Second, no direct measurements of free, bioavailable target hormones were performed. Indeed, as the use of heparinized lines in-ICU interferes with free thyroid hormone measurements [65] we also did not measure free T_4_ and T_3_ in the follow-up samples, and the complex, time-consuming methodology to measure bioavailable IGF-I [66] and free cortisol [13] does not allow analysis of such a large number of samples. Third, we have no information on ACTH concentrations as blood samples were not immediately stored on ice after collection, which precludes reliable ACTH measurements. Fourth, tissue hormone concentrations or metabolizing enzymes could not be evaluated. Fifth, our search for independent determinants of hormonal parameter concentrations at follow-up was only of exploratory nature and should not be overinterpreted. Of importance here, no information was available about the participants’ chronic nutritional status, whereas this could affect hormone concentrations as well [5, 38]. Finally, the studied patient cohort may be prone to selection bias. Non-survivors obviously could not be studied, whereas they are generally more severely ill, have a more complicated ICU trajectory, and overall show worse neuroendocrine disturbances in ICU than survivors [37, 67–70]. However, the three studied neuroendocrine axes also showed severe disturbances in the present cohort of survivors while in the ICU, representative of the impact of critical illness. The studied cohort was relatively enriched in sicker, long-stay patients as compared with the total EPaNIC cohort. Nevertheless, exclusion of patients with disabilities potentially confounding morbidity endpoints in the follow-up study, as well as availability of blood samples only from former ICU patients who were able to come to the hospital for participation in the study, may have introduced bias toward those with better physical capacity. Also, only a small number of patients were studied at the intermediate time points, which may have biased findings. That is why we only included these data for visual purposes without statistical analyses.

## Conclusions

Most critical illness-induced changes within the thyroid axis, the somatotropic axis and the adrenal axis had normalized 5 years after ICU admission, except for rT_3_ that remained supranormal resulting in persistently low T_3_/rT_3_ ratios, and for IGFBP3 concentrations that had increased to supranormal levels. In particular the residual long-term abnormality within the thyroid axis could be a harmful long-term neuroendocrine consequence of critical illness, contributing to the long-term physical impairment of former ICU patients. Whether targeting of this residual abnormality may improve long-term physical outcome remains to be investigated.

## Supplementary Information

**Additional file 1: Table S1.** Characteristics at the time of critical illness of patients who did not survive to 5-year follow-up versus 5-year survivors. Table with the characteristics upon ICU admission and ICU outcomes of patients who did not survive to 5-year follow-up versus 5-year survivors.

**Additional file 2: Table S2.** Characteristics at the time of critical illness of patients who survived to 5-years after ICU admission and did or did not participate in 5-year follow-up with provision of a blood sample. Table with the characteristics upon ICU admission and ICU outcomes of patients who survived to 5-years after ICU admission and did or did not participate in 5-year follow-up with provision of a blood sample.

**Additional file 3: Table S3.** Patient characteristics at the time of critical illness of former ICU patients who did or did not provide a serum sample at 5-year follow-up. Table with the characteristics upon ICU admission and ICU outcomes of patients who did or did not provide a serum sample at 5-year follow-up.

**Additional file 4: Table S4.** Determinants of serum concentrations of hormonal parameters of the thyroid, somatotropic or adrenal axis 5 years after critical illness. Table summarizing the results of multivariable analyses identifying factors independently associated with the serum concentrations of hormonal parameters of the thyroid, somatotropic or adrenal axis 5 years after critical illness.

**Additional file 5: Fig. S1.** Hormonal parameters of the thyroid, somatotropic and adrenal axis 5 years after critical illness in relation to duration of critical illness. Comparison of hormonal parameters at 5-year follow-up for patients who needed intensive care for fewer than 8 days or at least 8 days in bar graphs with univariable p values and as forest plot of β-estimates and 95% confidence intervals obtained with multivariable analyses adjusting for demographics.

## Data Availability

Data sharing will be considered only on a collaborative basis with the principal investigators, after evaluation of the proposed study protocol and statistical analysis plan.

## Abbreviations

ACTH: Adrenocorticotropic hormone
ADL: Activities of daily living
ANOVA: Analysis of variance
APACHE-II: Acute physiology and chronic health evaluation-II score
BMI: Body mass index
CBG: Cortisol-binding globulin
CI: Confidence interval
ELISA: Enzyme-linked immunosorbent assay
EN: Enteral nutrition
EPaNIC: Early parenteral nutrition completing enteral nutrition in adult critically ill patients
GHRH: Growth hormone-releasing hormone
ICU: Intensive care unit
IGF-I: Insulin-like growth factor-I
IGFBP1: Insulin-like growth factor binding protein 1
IGFBP3: Insulin-like growth factor binding protein 3
IQR: Interquartile range
IRMA: Immunoradiometric assay
PCS: Physical component score
PN: Parenteral nutrition
RCT: Randomized controlled trial
RIA: Radioimmunoassay
rT3: Reverse triiodothyronine
SF36: Medical Outcomes Report–Short Form 36 health-related quality-of-life questionnaire
T3: Triiodothyronine
T4: Tetraiodothyronine, thyroxine
TBG: Thyroxine-binding globulin
TBI: Traumatic brain injury
TSH: Thyroid-stimulating hormone
