# Supplementary material for: Persisting neuroendocrine abnormalities and their association with physical impairment 5 years after critical illness
Source: Crit Care. 2021 Dec 16;25:430. doi: 10.1186/s13054-021-03858-1 (PMC8675467; doi:10.1186/s13054-021-03858-1)
Supplement: Supplementary file 1 — Additional file 1: Table S1. Characteristics at the time of critical illness of patients who did not survive to 5-year follow-up versus 5-year survivors. Table with the characteristics upon ICU admission and ICU outcomes of patients who did not survive to 5-year follow-up versus 5-year survivors. [file 13054_2021_3858_MOESM1_ESM.docx]

**Additional Table 1: Characteristics at the time of critical illness of patients who did not survive to 5-year follow-up versus 5-year survivors**

| **Characteristic** | **5y survivor**  **(n=3214)** | **5y non-survivor**  **(n=1426)** | **P** |
| --- | --- | --- | --- |
| ***Characteristics upon ICU admission*** |  |  |  |
| Age (years), median (IQR) | 64 (53-73) | 71 (61-78) | <0.0001 |
| Sex (male), no (%) | 2075 (64.6) | 897 (62.9) | 0.27 |
| BMI (kg/m2), median (IQR) | 26.0 (23.4-29.2) | 25.1 (22.5-28.7) | <0.0001 |
| Randomized to early PN, no (%) | 1606 (50.0) | 706 (49.5) | 0.77 |
| Nutritional risk score ≥5, no (%) | 426 (13.3) | 437 (30.7) | <0.0001 |
| APACHE-II score first 24h, median (IQR) | 17 (13-28) | 28 (18-37) | <0.0001 |
| Emergency admission, no (%) | 1108 (34.5) | 818 (57.4) | <0.0001 |
| Admission diagnosis, no (%) |  |  | <0.0001 |
| Cardiac surgery | 2264 (70.4) | 554 (38.9) |  |
| Complicated abdominal or pelvic surgery | 155 (4.8) | 193 (13.5) |  |
| Transplantation | 246 (7.7) | 82 (5.8) |  |
| Trauma, burns or reconstructive surgery | 146 (4.5) | 60 (4.2) |  |
| Complicated pulmonary or esophageal surgery | 66 (2.1) | 103 (7.2) |  |
| Respiratory disease | 46 (1.4) | 97 (6.8) |  |
| Complicated vascular surgery | 76 (2.4) | 58 (4.1) |  |
| Gastroenterologic or hepatic disease | 50 (1.6) | 66 (4.6) |  |
| Complicated neurosurgery | 60 (1.9) | 55 (3.9) |  |
| Hematological or oncological disease | 2 (0.1) | 32 (2.2) |  |
| Neurological disease | 11 (0.3) | 6 (0.4) |  |
| Cardiovascular disease | 4 (0.1) | 14 (1.0) |  |
| Renal disease | 6 (0.2) | 12 (0.8) |  |
| Neurological presentation of medical disease | 15 (0.5) | 14 (1.0) |  |
| Metabolic disorder | 4 (0.1) | 2 (0.1) |  |
| Other | 63 (2.0) | 78 (5.5) |  |
| History of diabetes, no (%) | 494 (15.4) | 314 (22.0) | <0.0001 |
| History of malignancy, no (%) | 400 (12.5) | 494 (34.6) | <0.0001 |
| Pre-admission dialysis, no (%) | 25 (0.8) | 44 (3.1) | <0.0001 |
| Sepsis upon admission, no (%) | 493 (15.3) | 522 (36.6) | <0.0001 |
| ***ICU outcomes*** |  |  |  |
| New infection in ICU, no (%) | 587 (18.3) | 549 (38.5) | <0.0001 |
| New need of dialysis, no (%) | 115 (3.6) | 244 (17.1) | <0.0001 |
| Duration of mechanical ventilation (days), median (IQR) | 2 (1-3) | 3 (2-11) | <0.0001 |
| Corticosteroid treatment, no (%) | 577 (18.0) | 583 (40.9) | <0.0001 |
| Duration of corticosteroid treatment (days), median (IQR) | 0 (0-0) | 0 (0-4) | <0.0001 |
| ICU length of stay (days), median (IQR) | 3 (2-6) | 6 (3-15) | <0.0001 |
| Hospital length of stay (days), median (IQR) | 13 (9-23) | 21 (12-41) | <0.0001 |

APACHE-II score: acute physiology and chronic health evaluation-II score, ICU: intensive care unit, IQR: interquartile range, PN: parenteral nutrition.
