# Supplementary material for: Persisting neuroendocrine abnormalities and their association with physical impairment 5 years after critical illness
Source: Crit Care. 2021 Dec 16;25:430. doi: 10.1186/s13054-021-03858-1 (PMC8675467; doi:10.1186/s13054-021-03858-1)
Supplement: Supplementary file 2 — Additional file 2: Table S2. Characteristics at the time of critical illness of patients who survived to 5-years after ICU admission and did or did not participate in 5-year follow-up with provision of a blood sample. Table with the characteristics upon ICU admission and ICU outcomes of patients who survived to 5-years after ICU admission and did or did not participate in 5-year follow-up with provision of a blood sample. [file 13054_2021_3858_MOESM2_ESM.docx]

**Additional Table 2: Characteristics at the time of critical illness of patients who survived to 5-years after ICU admission and did or did not participate in 5-year follow-up with provision of a blood sample**

| **Characteristic** | **Participants 5y neuroendocrine evaluation**  **(n=436)** | **Other 5y survivors**  **(n=2778)** | **P** |
| --- | --- | --- | --- |
| ***Characteristics upon ICU admission*** |  |  |  |
| Age (years), median (IQR) | 56 (46-64) | 66 (55-74) | <0.0001 |
| Sex (male), no (%) | 302 (69.3) | 1773 (63.8) | 0.025 |
| BMI (kg/m2), median (IQR) | 25.7 (23.1-28.4) | 26.0 (23.4-29.3) | 0.041 |
| Randomized to early PN, no (%) | 222 (50.9) | 1384 (49.8) | 0.67 |
| Nutritional risk score ≥5, no (%) | 70 (16.1) | 356 (12.8) | 0.069 |
| APACHE-II score first 24h, median (IQR) | 26 (16-33) | 17 (13-26) | <0.0001 |
| Emergency admission, no (%) | 271 (62.2) | 837 (30.1) | <0.0001 |
| Admission diagnosis, no (%) |  |  | <0.0001 |
| Cardiac surgery | 168 (38.5) | 2096 (75.5) |  |
| Complicated abdominal or pelvic surgery | 44 (10.1) | 111 (4.0) |  |
| Transplantation | 76 (17.4) | 170 (6.1) |  |
| Trauma, burns or reconstructive surgery | 57 (13.1) | 89 (3.2) |  |
| Complicated pulmonary or esophageal surgery | 12 (2.8) | 54 (1.9) |  |
| Respiratory disease | 9 (2.1) | 37 (1.3) |  |
| Complicated vascular surgery | 19 (4.4) | 57 (2.1) |  |
| Gastroenterologic or hepatic disease | 9 (2.1) | 41 (1.5) |  |
| Complicated neurosurgery | 16 (3.7) | 44 (1.6) |  |
| Hematological or oncological disease | 2 (0.5) | 0 (0.0) |  |
| Neurological disease | 2 (0.5) | 9 (0.3) |  |
| Cardiovascular disease | 3 (0.7) | 1 (0.0) |  |
| Renal disease | 1 (0.2) | 5 (0.2) |  |
| Neurological presentation of medical disease | 2 (0.5) | 13 (0.5) |  |
| Metabolic disorder | 1 (0.2) | 3 (0.1) |  |
| Other | 15 (3.4) | 48 (1.7) |  |
| History of diabetes, no (%) | 47 (10.8) | 447 (16.1) | 0.0029 |
| History of malignancy, no (%) | 55 (12.6) | 345 (12.4) | 0.90 |
| Pre-admission dialysis, no (%) | 3 (0.7) | 22 (0.8) | 0.81 |
| Sepsis upon admission, no (%) | 118 (27.1) | 375 (13.5) | <0.0001 |
| ***ICU outcomes*** |  |  |  |
| New infection in ICU, no (%) | 145 (33.3) | 442 (15.9) | <0.0001 |
| New need of dialysis, no (%) | 38 (8.7) | 77 (2.8) | <0.0001 |
| Duration of mechanical ventilation (days), median (IQR) | 3 (1-8) | 2 (1-3) | <0.0001 |
| Corticosteroid treatment, no (%) | 148 (33.9) | 429 (15.4) | <0.0001 |
| Duration of corticosteroid treatment (days), median (IQR) | 0 (0-2) | 0 (0-0) | <0.0001 |
| ICU length of stay (days), median (IQR) | 5 (2-13) | 3 (1-5) | <0.0001 |
| Hospital length of stay (days), median (IQR) | 21 (11-35) | 13 (9-21) | <0.0001 |

APACHE-II score: acute physiology and chronic health evaluation-II score, ICU: intensive care unit, IQR: interquartile range, PN: parenteral nutrition.
