# Supplementary material for: Persisting neuroendocrine abnormalities and their association with physical impairment 5 years after critical illness
Source: Crit Care. 2021 Dec 16;25:430. doi: 10.1186/s13054-021-03858-1 (PMC8675467; doi:10.1186/s13054-021-03858-1)
Supplement: Supplementary file 3 — Additional file 3: Table S3. Patient characteristics at the time of critical illness of former ICU patients who did or did not provide a serum sample at 5-year follow-up. Table with the characteristics upon ICU admission and ICU outcomes of patients who did or did not provide a serum sample at 5-year follow-up. [file 13054_2021_3858_MOESM3_ESM.docx]

**Additional Table 3: Patient characteristics at the time of critical illness of former ICU patients who did or did not provide a serum sample at 5-year follow-up**

| **Characteristic** | **Serum sample**  **(n=436)** | **No serum sample**  **(n=238)** | **P** |
| --- | --- | --- | --- |
| ***Characteristics upon ICU admission*** |  |  |  |
| Age (years), median (IQR) | 56 (46-64) | 69 (60-77) | <0.0001 |
| Sex (male), no (%) | 302 (69.3) | 148 (62.2) | 0.063 |
| BMI (kg/m2), median (IQR) | 25.7 (23.1-28.4) | 25.8 (22.6-29.3) | 0.76 |
| Randomized to early PN, no (%) | 222 (50.9) | 108 (45.4) | 0.16 |
| Nutritional risk score ≥5, no (%) | 70 (16.1) | 59 (24.8) | 0.0066 |
| APACHE-II score first 24h, median (IQR) | 26 (16-33) | 22 (16-33) | 0.68 |
| Emergency admission, no (%) | 271 (62.2) | 106 (44.5) | <0.0001 |
| Admission diagnosis, no (%) |  |  | 0.0005 |
| Cardiac surgery | 168 (38.5) | 133 (55.9) |  |
| Complicated abdominal or pelvic surgery | 44 (10.1) | 13 (5.5) |  |
| Transplantation | 76 (17.4) | 36 (15.1) |  |
| Trauma, burns or reconstructive surgery | 57 (13.1) | 9 (3.8) |  |
| Complicated pulmonary or esophageal surgery | 12 (2.8) | 7 (2.9) |  |
| Respiratory disease | 9 (2.1) | 3 (1.3) |  |
| Complicated vascular surgery | 19 (4.4) | 14 (5.9) |  |
| Gastroenterologic or hepatic disease | 9 (2.1) | 6 (2.5) |  |
| Complicated neurosurgery | 16 (3.7) | 5 (2.1) |  |
| Hematological or oncological disease | 2 (0.5) | 0 (0.0) |  |
| Neurological disease | 2 (0.5) | 0 (0.0) |  |
| Cardiovascular disease | 3 (0.7) | 0 (0.0) |  |
| Renal disease | 1 (0.2) | 1 (0.4) |  |
| Neurological presentation of medical disease | 2 (0.5) | 1 (0.4) |  |
| Metabolic disorder | 1 (0.2) | 1 (0.4) |  |
| Other | 15 (3.4) | 9 (3.8) |  |
| History of diabetes, no (%) | 47 (10.8) | 52 (21.9) | 0.0001 |
| History of malignancy, no (%) | 55 (12.6) | 51 (21.4) | 0.0031 |
| Pre-admission dialysis, no (%) | 3 (0.7) | 0 (0.0) | 0.10 |
| Sepsis upon admission, no (%) | 118 (27.1) | 58 (24.4) | 0.44 |
| ***ICU outcomes*** |  |  |  |
| New infection in ICU, no (%) | 145 (33.3) | 85 (35.7) | 0.52 |
| New need of dialysis, no (%) | 38 (8.7) | 19 (8.0) | 0.74 |
| Duration of mechanical ventilation (days), median (IQR) | 3 (1-8) | 3 (2-8) | 0.80 |
| Corticosteroid treatment, no (%) | 148 (33.9) | 75 (31.5) | 0.52 |
| Duration of corticosteroid treatment (days), median (IQR) | 0 (0-2) | 0 (0-2) | 0.59 |
| ICU length of stay (days), median (IQR) | 5 (2-13) | 6 (3-13) | 0.12 |
| Hospital length of stay (days), median (IQR) | 21 (11-35) | 22 (13-37) | 0.21 |

APACHE-II score: acute physiology and chronic health evaluation-II score, ICU: intensive care unit, IQR: interquartile range, PN: parenteral nutrition.
