# Supplementary material for: Persisting neuroendocrine abnormalities and their association with physical impairment 5 years after critical illness
Source: Crit Care. 2021 Dec 16;25:430. doi: 10.1186/s13054-021-03858-1 (PMC8675467; doi:10.1186/s13054-021-03858-1)
Supplement: Supplementary file 4 — Additional file 4: Table S4. Determinants of serum concentrations of hormonal parameters of the thyroid, somatotropic or adrenal axis 5 years after critical illness. Table summarizing the results of multivariable analyses identifying factors independently associated with the serum concentrations of hormonal parameters of the thyroid, somatotropic or adrenal axis 5 years after critical illness. [file 13054_2021_3858_MOESM4_ESM.docx]

**Additional Table 4: Determinants of serum concentrations of hormonal parameters of the thyroid, somatotropic or adrenal axis 5 years after critical illness**

| **Variable** | **TSH** | **T4** | **T3** | **rT3** | **T3/rT3** | **TBG** | **GH** | **IGF-I** | **IGFBP3** | **IGFBP1** | **Cortisol** | **Free Cortisol** | **CBG** | **Albumin** |
| --- | --- | --- | --- | --- | --- | --- | --- | --- | --- | --- | --- | --- | --- | --- |
| Age at 5-year follow-up |  |  | - | + | - |  |  | - |  | + |  | + | - | - |
| Male sex | + | - |  |  |  |  | - |  | - | - |  |  | - |  |
| BMI at 5-year follow-up |  |  |  |  |  |  | - |  | - | - | - |  |  |  |
| History of diabetes |  |  |  |  |  |  |  |  |  |  |  |  |  |  |
| History of malignancy |  |  |  |  |  |  |  |  | + |  |  |  |  |  |
| Sepsis upon ICU admission |  |  |  |  |  |  |  |  |  |  |  |  |  |  |
| High risk of malnutrition * | + |  |  |  |  |  |  |  |  |  |  |  |  |  |
| APACHE-II first 24 hours |  |  |  |  |  |  | + |  |  | + |  |  |  |  |
| Diagnosis requiring ICU admission ** |  |  |  |  |  |  |  |  |  |  |  |  |  |  |
| Cardiovascular |  |  |  |  |  |  | + |  |  | + | + | + |  |  |
| Abdominal |  |  |  |  |  |  |  |  |  |  |  |  |  |  |
| Respiratory |  |  |  |  |  |  |  |  |  |  |  |  |  |  |
| Neurological | - |  |  |  |  |  |  |  |  | - | - | - |  |  |
| Transplant | + | + |  | + | - |  |  |  |  | + |  |  |  |  |
| Trauma, burns, reconstructive surgery |  |  |  |  |  |  | - |  |  |  |  |  |  |  |
| Other medical disease | + | - |  |  |  |  |  |  |  |  |  |  |  |  |
| Randomization to late-PN |  |  |  |  |  |  |  |  |  |  |  |  |  |  |
| Prolonged need of intensive care *** |  |  |  |  |  |  |  |  |  |  |  |  |  |  |
| Medication at 5-year follow-up |  |  |  |  |  |  |  |  |  |  |  |  |  |  |
| Insulin therapy |  |  |  |  |  |  |  |  |  |  |  |  |  |  |
| Corticosteroids |  |  |  |  |  |  |  | + | + |  |  |  |  |  |
| Anticoagulants |  |  | - | + | - |  |  |  |  |  |  |  |  |  |
| Betablockers |  |  |  | + | - |  |  |  |  |  |  |  |  |  |
| Antidepressants | + | - |  |  |  |  |  | - |  |  | - | - |  |  |
| Antipsychotics |  |  |  |  |  |  |  |  |  |  | + | + | + |  |
| Oral antidiabetic medication |  |  |  |  |  |  |  |  |  |  |  |  |  |  |
| Statins |  |  |  |  |  |  | - |  |  | - |  |  |  |  |
| Hospital readmission for any reason |  |  |  |  |  |  |  | + |  |  | - | - |  |  |

* Nutritional Risk Screening score 5 or higher; ** “Cardiovascular (cardiovascular surgery or disease)”, “Abdominal (complicated abdominal or pelvic surgery, or gastroenterologic or hepactic disease)”, “Respiratory (complicated pulmonary or esophageal surgery or respiratory disease)”, “Neurological (complicated neurosurgery, neurological disease, or neurological presentation of medical disease)”, “Transplantation”, “Trauma, burns, reconstructive surgery”, “Other medical disease” (renal, metabolic, hemato/onco, other); ICU length of stay of at least 8 days. “+” and “-“ symbols or blue and yellow highlighting point to positive and negative independent associations with the hormonal parameter in the column. APACHE: acute physiology and health evaluation score.
