# Supplementary material for: Persisting neuroendocrine abnormalities and their association with physical impairment 5 years after critical illness
Source: Crit Care. 2021 Dec 16;25:430. doi: 10.1186/s13054-021-03858-1 (PMC8675467; doi:10.1186/s13054-021-03858-1)
Supplement: Supplementary file 5 — Additional file 5: Fig. S1. Hormonal parameters of the thyroid, somatotropic and adrenal axis 5 years after critical illness in relation to duration of critical illness. Comparison of hormonal parameters at 5-year follow-up for patients who needed intensive care for fewer than 8 days or at least 8 days in bar graphs with univariable p values and as forest plot of β-estimates and 95% confidence intervals obtained with multivariable analyses adjusting for demographics. [file 13054_2021_3858_MOESM5_ESM.docx]

**Additional Figure 1: Hormonal parameters of the thyroid, somatotropic and adrenal axis 5 years after critical illness in relation to duration of critical illness**

Patients who received thyroid hormone treatment in ICU or were on chronic thyroid hormone treatment at follow-up were excluded for the analysis of the thyroid axis, patients on chronic GHRH or somatostatin analogue treatment at follow-up were excluded for the analysis of the somatotropic axis and patients on corticosteroid treatment in ICU or on chronic corticosteroid treatment at follow-up were excluded for analysis of the adrenal axis. Panel A shows the univariable analyses comparing hormonal parameters 5 years after critical illness of former ICU patients who needed intensive care for shorter than 8 days versus those who needed intensive care for at least 8 days. Data are shown as mean and standard error of the mean. TSH, growth hormone (GH), IGFBP1, and total and free cortisol concentrations were square root-square root transformed and rT_3_ and T_3_/rT_3_ were square root transformed to obtain a near normal distribution, allowing t test. Y-axes were transformed back to original values. Panel B depicts the corresponding multivariable linear regression analyses showing β-estimates and 95% confidence intervals for patients who needed intensive care for at least 8 days as compared with those who needed intensive care for a shorter time, adjusting for demographics (sex, and age and BMI at 5-year follow-up). The latter was necessary as the two patient groups were not balanced for demographics, with significant differences in sex and age, as shown in the table below.

|  | **Thyroid axis** | | | **Somatotropic axis** | | | **Adrenal axis** | | |
| --- | --- | --- | --- | --- | --- | --- | --- | --- | --- |
|  | **<8 days** | **At least 8 days** | **P** | **<8 days** | **At least 8 days** | **P** | **<8 days** | **At least 8 days** | **P** |
|  | n=241 | n=162 |  | n=264 | n=169 |  | n=193 | n=92 |  |
| Male sex | 162 (67.2) | 123 (75.9) | 0.057 | 172 (65.2) | 128 (75.7) | 0.018 | 133 (68.9) | 77 (83.7) | 0.0064 |
| Age | 62 (52-70) | 60 (50-66) | 0.030 | 62 (52-70) | 60 (49-67) | 0.026 | 64 (53-73) | 59 (49-66) | 0.0027 |
| BMI | 27.2 (23.9-30.1) | 27.3 (24.4-30.9) | 0.32 | 27.3 (24.1-30.3) | 27.2 (24.4-30.9) | 0.57 | 27.7 (24.5-30.6) | 27.5 (24.4-30.9) | 0.82 |
